# “It seemed I was having a conversation with him”: Posthumous Dignity Therapy case series

**DOI:** 10.1017/S1478951525000173

**Published:** 2025-02-28

**Authors:** Miguel Julião, Carolina Simões, Harvey Max Chochinov

**Affiliations:** 1 Department of Palliative Medicine, Equipa Comunitária de Suporte em Cuidados Paliativos, ULS Amadora/Sintra, Amadora, Portugal; 2 Department of Psychiatry, University of Manitoba, Cancer Care Manitoba Research Institute, Winnipeg, MB, Canada

**Keywords:** Dignity Therapy, bereavement, Dignity Therapy by proxy, caregivers, palliative care

## Abstract

**Objectives:**

Dignity Therapy (DT) is a brief psychotherapeutic intervention designed to address the psychosocial and spiritual needs of terminally ill patients. Research demonstrates DT’s efficacy in reducing dignity-related distress and alleviating psychosocial symptoms like depression and anxiety in terminally ill patients. Its application has been extended to nonterminal patients with chronic conditions, mental health challenges, and children nearing the end of life, with promising results. DT also benefits families and caregivers, promoting emotional resilience and facilitating grieving. However, the potential for proxy applications, such as posthumous DT (p-DT) – conducted by relatives after a patient’s death or on behalf of individuals unable to participate – remains underexplored.

**Methods:**

A case series report.

**Results:**

This case series examines 3 relatives who engaged in p-DT, highlighting its feasibility and potential benefits.

**Significance of results:**

Findings suggest p-DT may serve as a valuable tool for bereavement support, warranting further research to expand its scope and accessibility.

## Introduction

Dignity Therapy (DT) is a brief psychotherapeutic intervention designed to address psychosocial and spiritual issues facing terminally ill patients. It allows patients nearing death to share their memories, wisdom, and hopes with loved ones through the creation of a legacy document, hence fostering a sense of purpose and generativity. This helps patients leave a lasting mark, while supporting the well-being of their bereaved loved ones (Chochinov et al. 2005).

DT has been shown to reduce dignity-related distress, enhance meaning, and alleviate psychosocial distress such as depression and anxiety, particularly for adults with life-threatening illnesses (Chochinov et al. 2011; Julião et al. 2014; Cuevas et al. 2021; Johnston et al. 2023). Applications of DT have expanded to diverse populations, including nonterminally ill patients with chronic conditions, mental disorders (Avery and Savitz 2011; Avery and Baez 2012; Lubarsky and Avery 2016; Julião 2019), and dying children and adolescents (Julião et al. 2020a; Julião et al. 2020b); the results thus far appear promising.

**Figure 1.**
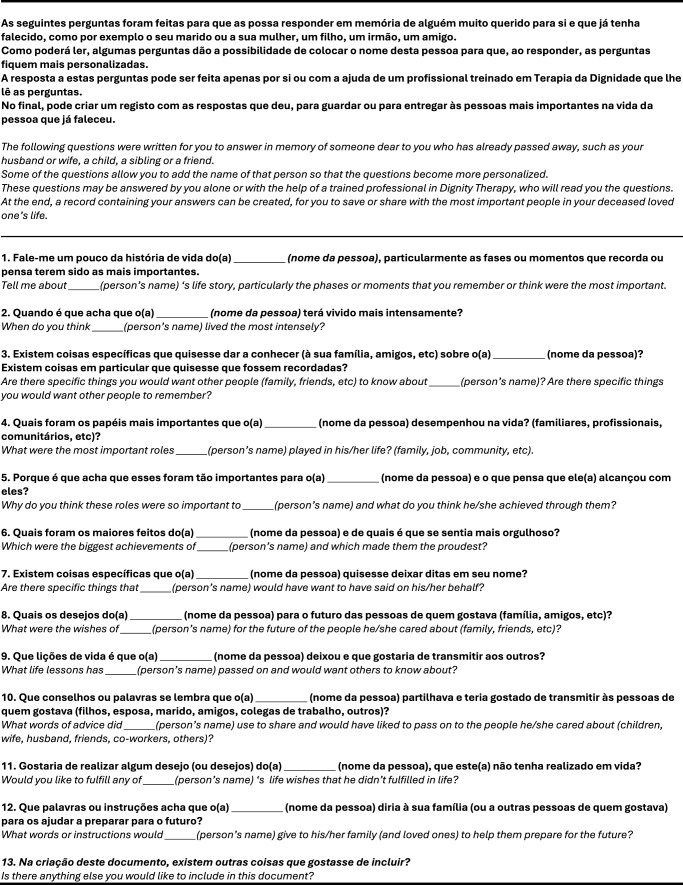
The European Portuguese posthumous schedule of questions.

DT has also proven beneficial for families and caregivers, who receive the generativity documents, helping ease the grieving process, facilitating better communication and transgenerational connectedness (Grijó et al. 2021; Julião et al. 2023). However, little has been done to explore whether DT can be applied by proxy (Fig. 1); that is, whether it can be undertaken by someone following the patient’s death (i.e., posthumous DT or p-DT [Julião et al. 2023]) or in circumstances where the patient – such an infant, young child, or adult lacking capacity – is unable to engage in DT themselves.

## Case series

Three family members of patients of Dr. Julião´s who had died in home-based palliative care were invited to take part. They were given an explanation of p-DT, and a framework of questions designed to be applied posthumously (Julião et al. 2023; Fig. 1). They had the option to engage in p-DT with the support of a dignity therapist or independently. They were also told that neither written answers nor creating a physical generativity document was mandatory, and that the time required to complete this was flexible and at their discretion. Following this process, participants were contacted by phone to elicit their impressions. The content of these interviews was audio recorded and transcribed.

Participants included a 43-year-old man whose father had died 1 year ago; an 81-year-old woman whose husband had died 3 years ago; and a 60-year-old man whose wife died 5 years ago. All 3 chose to perform p-DT independently; 2 opted to write their responses into a generativity document, which they kept for personal reflection and revisit at their convenience. The youngest participant – the son whose father had died – chose to answer the questions aloud without creating a written document. The duration of this intervention varied, spanning from 3 days to 1 week.

## The 3 participants recorded impressions are presented below

### A wife’s perspective

*When I first heard about Posthumous Dignity Therapy, I was hesitant. My husband had passed away from end-stage pancreatic cancer, and the pain of his absence was still raw. The idea of answering questions based on his legacy felt both comforting and daunting. But as I began the process, something truly remarkable happened.*

*Sitting down to answer and writing the questions, I felt an overwhelming sense of peace. It was as though he was there with me, guiding my thoughts and helping me find the right words. The questions brought back memories of his kindness, his dreams, and even his quirks that made me smile. For the first time since his passing, I felt his presence – not just in the memories, but in the room with me.*

*Answering the questions wasn’t easy at first; there were tears and moments where I had to pause. But it allowed me to reconnect with the essence of who he was. It wasn’t just about recounting his life – it was about honouring him, carrying forward the love and wisdom he left behind, and finding a way to share that with our son.*

*Most importantly, the process helped me in my grief. It shifted my focus from the pain of his absence to the beauty of his legacy. I could feel his support, as if he were sitting beside me, whispering, “You’ve got this.” By the end, I wasn’t just answering questions – it seemed I was having a conversation with him, one that transcended his physical absence.*

*This experience has brought me an immense sense of closure and peace. It helped me realize that even though he is no longer here, his presence remains in the love we shared and the life we built together. Posthumous Dignity Therapy gave me a way to connect with him in a deeply personal and meaningful way, reminding me that love and legacy endure, even in the face of loss.*

### A husband’s journey

*When my wife passed away after her battle with breast cancer, I felt as though the world had stopped. The silence in our home was deafening, filled with all the things I wished I had said and done. So, when I was introduced to Posthumous Dignity Therapy, I wasn’t sure what to expect. I only knew that I wanted to feel closer to her again, even if just for a moment and I had to trust Dr. Julião that took care of her so well and closely until her death.*

*The process lasted three days, and it was unlike anything I could have imagined. As I worked through the legacy document, it felt like she was right there with me. I could almost hear her voice as I recalled her hopes, dreams, and the little things that made her who she was, and everything she ever did for others. There were moments when the memories overwhelmed me, and I couldn’t stop crying, but it was not worrying. But then there were times when I found myself laughing, remembering her humor and the way she could light up a room with her smile.*

*What surprised me most was how much the therapy felt like a two-way conversation. Although she wasn’t physically present, answering the questions gave me the chance to say things I had never said to her while she was alive. I apologized for my regrets, told her how much I loved her, and even talked about the plans and trips we never got to fulfil. It was deeply emotional but also profoundly healing.*

*One of her dreams was to visit Paris – a trip we had always talked about but never managed to take. As I completed the therapy, I felt a quiet resolve to honor her by making that journey myself. A few months later, I travelled to Paris, carrying a small token of hers with me. It was as though she was walking beside me, experiencing the beauty and wonder of the place she had always dreamed of seeing.*

*Posthumous Dignity Therapy didn’t just help me process my grief – it gave me the opportunity to reconnect with my wife in a way I didn’t think was possible. It allowed me to feel her presence, to laugh, cry, and, most importantly, to find peace. Through this process, I realized that her love and dreams continue to guide me, even now. And for that, I am endlessly grateful.*

### A son’s journey

*Losing my dad to advanced prostate cancer left a hole in my life that nothing seemed to fill. We were very close… very close to each other. He was my rock, my guide, and suddenly he was gone, leaving behind so many things unsaid, so many words I wished I could have spoken. When I was introduced to Posthumous Dignity Therapy, I wasn’t sure if it would help. But deep down, I knew I needed something – something to bring me closer to him, even though he wasn’t physically here.*

*At first, I felt a knot in my stomach as I sat down to answer the questions. I felt afraid. I was afraid of not knowing what to say or write, afraid that I wouldn’t be able to find the words to truly express everything I had kept inside all these years. The thought of speaking to my father – who I could no longer touch or hear – felt overwhelming. But I pushed through, and with each question, I found myself unlocking a bit more courage.*

*In the beginning, I stumbled over my words. I paused, sometimes choked on my emotions, wondering if my dad could somehow hear me, or if this process was even possible. But as I continued, the fear began to fade. I realized that this was my opportunity to speak freely to him – to tell him all the things I’d never gotten the chance to say before his illness took him away. I told him how much I admired him, how grateful I was for everything he had done for me, and how I had always wished I had expressed that more often.*

*The words started flowing more naturally, and soon, I felt like he was listening. There was no more fear, only a sense of peace. It was as if I had finally opened the door to the conversation we never had, and he was there with me in spirit, guiding me. I could tell him about my life now, how I was coping without him, how much I missed him along with my mother and his granddaughters, and even share some of the things I was proud of doing that I knew he would have been proud of too.*

*Through this process, I realized that it wasn’t just about saying goodbye – it was about finding closure. It was about telling him the things I should have said when he was still here, things I hadn’t had the courage to say. But most of all, it was about honoring him, not just in memory, but in living on with the lessons and love he had given me.*

*Posthumous Dignity Therapy gave me a space to grieve, but also the opportunity to heal. It allowed me to speak to my dad with a courage I hadn’t known I had, and it left me with a sense of peace – peace knowing that, somehow, he’d heard me, and that he would always be with me in the lessons he taught and the love we shared.*

## Discussion

To the best of our knowledge, this is the first time bereaved individuals have reported their impressions of DT performed posthumously, that is by way of proxy. Their overwhelmingly positive response suggests this approach holds promise as a novel bereavement intervention.

While the 3 participants in this case series chose to engage in p-DT independently, it is certainly conceivable others might wish to have this process facilitated by a qualified therapist. Knowing both modalities are workable adds to the potential feasibility of this approach. It should also be noted that while 2 participants seemed to value the creation of a tangible legacy document, 1 (the bereft son) appreciated the opportunity to verbally respond to the framework of questions without any documentation; and reported a deep sense of connectedness, meaning and catharsis.

None of the participants completed p-DT in a single session. This demonstrates the plasticity of this therapeutic process and highlights the importance of maintaining contact with participants to monitor their emotional state. Since this process can be highly emotionally evocative, psychological support may sometimes be necessary. That being said, none of the participants reported feeling emotional dysregulation or the need for additional support.

The length of time from the patient’s death across this case series spanned 5 years. This suggests p-DT may be applicable at different time intervals relative to when the patient died, depending on the bereft individual’s state of mind and perhaps existential readiness. Two of the 3 participants in this case series were unable to have proper funerals due to the COVID-19 pandemic. In both instances, p-DT offered a sense of closure and a tangible way of honoring the deceased.

While all these cases occurred posthumously, there is no reason to think it might not have applications in other circumstances where the patient does not, or no longer has, a voice. One can imagine DT by proxy protocols being designed for family members and loved ones of critically ill infants; children unable to give voice to their story; or adults whose cognitive capacities are no longer compatible with being able to construct their own legacy.

## Conclusion

While the numbers of participants in this case series are small, the idea of DT by proxy is large. Future research is needed to explore how this might inform and transform the experience of bereavement; or how it might affect families and loved ones caring and supporting those unable to tell their own unique story.

## References

[ref1] Avery JD and Baez MA (2012) Dignity therapy for major depressive disorder: A case report. *Journal of Palliative Medicine* 15(5), 509–509. doi:10.1089/jpm.2011.0522 22577783

[ref2] Avery JD and Savitz AJ (2011) A novel use of dignity therapy. *American Journal of Psychiatry* 168(12), 1340–1340. doi:10.1176/appi.ajp.2011.11030444 22193678

[ref3] Chochinov HM, Hack T, Hassard T, et al. (2005) Dignity therapy: A novel psychotherapeutic intervention for patients near the end of life. *Journal of Clinical Oncology* 23(24), 5520–5525. doi:10.1200/JCO.2005.08.391 16110012

[ref4] Chochinov HM, Kristjanson LJ, Breitbart W, et al. (2011) Effect of dignity therapy on distress and end-of-life experience in terminally ill patients: A randomised controlled trial. *The Lancet Oncology* 12(8), 753–762. doi:10.1016/S1470-2045(11)70153-X 21741309 PMC3185066

[ref5] Cuevas PE, Davidson P, Mejilla J, et al. (2021) Dignity therapy for end-of-life care patients: A literature review. *Journal of Patient Experience* 8, 2374373521996951. doi:10.1177/2374373521996951 PMC8205385 34179373

[ref6] Grijó L, Tojal C and Rego F (2021) Effects of dignity therapy on palliative patients’ family members: A systematic review. *Palliative and Supportive Care* 19(5), 605–614. doi:10.1017/S147895152100033X 33818358

[ref7] Johnston B, Dönmez CF and Julião M (2023) Effectiveness of dignity therapy in the context of culturally competent care in people with palliative care needs: A systematic review of systematic reviews. *Current Opinion in Supportive & Palliative Care* 17(3), 186–192. doi:10.1097/SPC.0000000000000664 37428208

[ref8] Julião M (2019) The use of dignity therapy beyond terminal illness: A case report. *Psychosomatics* 60(1), 101. doi:10.1016/j.psym.2018.05.004 29891255

[ref9] Julião M, Antunes B, Santos A, et al. (2020a) Adapting the Portuguese dignity question framework for adolescents: Ages 10–18. *Palliative and Supportive Care* 18(2), 199–205. doi:10.1017/S1478951519000798 31559945

[ref10] Julião M, Chochinov H, Antunes B, et al. (2023) The European Portuguese posthumous dignity therapy schedule of questions: Initial development and validation. *Palliative and Supportive Care* 21(1), 74–82. doi:10.1017/S1478951522000396 35586874

[ref11] Julião M, Oliveira F, Nunes B, et al. (2014) Efficacy of dignity therapy on depression and anxiety in Portuguese terminally ill patients: A phase II randomized controlled trial. *Journal of Palliative Medicine* 17(6), 688–695. doi:10.1089/jpm.2013.0567 24735024

[ref12] Julião M, Santos A, Albuquerque S, et al. (2020b) Operationalizing dignity therapy for adolescents. *Palliative and Supportive Care* 18(5), 626–631. doi:10.1017/S1478951520000589 32729449

[ref13] Lubarsky KE and Avery JD (2016) Dignity therapy for alcohol use disorder. *American Journal of Psychiatry* 173(1), 90–90. doi:10.1176/appi.ajp.2015.15070851 26725343

